# Supplementary material for: Coupling machine learning and crop modeling improves crop yield prediction in the US Corn Belt
Source: Sci Rep. 2021 Jan 15;11:1606. doi: 10.1038/s41598-020-80820-1 (PMC7810832; doi:10.1038/s41598-020-80820-1)
Supplement: Supplementary file 1 — Supplementary Information [file 41598_2020_80820_MOESM1_ESM.docx]

**Coupling Machine Learning and Crop Modeling Improves Crop Yield Prediction in the US Corn Belt**

Mohsen Shahhosseini^1^, Guiping Hu^1*^, Isaiah Huber^2^, Sotirios V. Archontoulis^2^_,_

^1^ Department of Industrial and Manufacturing Systems Engineering, Iowa State University, Ames, Iowa, USA

^2^ Department of Agronomy, Iowa State University, Ames, Iowa, USA

* Corresponding author e-mail: gphu@iastate.edu

Table S1: Test data summary statistics

| *Test Year* | *Average (kg/ha)* | *Standard deviation (kg/ha)* | *Number of counties* |
| --- | --- | --- | --- |
| 2018 | 12,238 | 1337 | 257 |
| 2017 | 11,903 | 1457 | 278 |
| 2012 | 6,646 | 2410 | 273 |

Summary statistics of some of the best performing designed ML models are shown in Figure S1. The probability density functions of these models are depicted along with the ground truth. This plot suggests that the ML models can maintain the same probability density function to some extent.


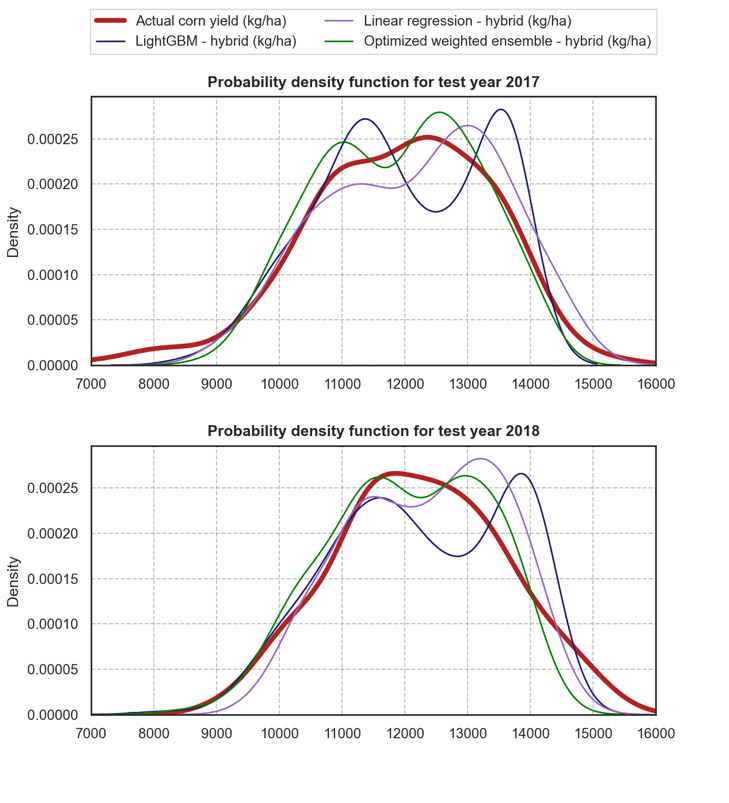


Figure S1: Probability density function of ground truth compared to some of designed ML models
